# High Frequency of *Enterocytozoon bieneusi* Genotype WL12 Occurrence among Immunocompromised Patients with Intestinal Microsporidiosis

**DOI:** 10.3390/jof7030161

**Published:** 2021-02-24

**Authors:** Mariem Messaoud, Salma Abbes, Mayssa Gnaien, Yasmine Rebai, Aicha Kallel, Sana Jemel, Ghaya Cherif, Mohamed Amine Skhairia, Sonia Marouen, Najla Fakhfekh, Helmi Mardassi, Slaheddine Belhadj, Sadri Znaidi, Kalthoum Kallel

**Affiliations:** 1 Laboratoire de Parasitologie et Mycologie, UR17SP03, La Rabta Hospital, Tunis 1007, Tunisia; messaoudmariem@yahoo.fr (M.M.); sel_salma@yahoo.fr (S.A.); aicha@elloumi.net (A.K.); jemelsana.benayed@gmail.com (S.J.); ghaya_cherif@yahoo.fr (G.C.); sonia.marouen@gmail.com (S.M.); najlafakhfakh@yahoo.fr (N.F.); slaheddinebelhadj@gmail.com (S.B.); 2 Institut Pasteur de Tunis, University of Tunis El Manar, Laboratoire de Microbiologie Moléculaire, Vaccinologie et Développement Biotechnologique, Tunis 1002, Tunisia; mayssagn1@gmail.com (M.G.); yassmine.rebai@gmail.com (Y.R.); med_amine_skhairia@outlook.fr (M.A.S.); helmi.merdassi@pasteur.tn (H.M.); 3 Institut Pasteur, INRA, Département Mycologie, Unité Biologie et Pathogénicité Fongiques, 75015 Paris, France

**Keywords:** *Enterocytozoon bieneusi*, *Encephalitozoon spp*, immunocompromised patients, HIV, emerging infectious diseases, genotype WL12, molecular diagnosis

## Abstract

Microsporidiosis is an emerging opportunistic infection causing severe digestive disorders in immunocompromised patients. The aim of this study was to investigate the prevalence of intestinal microsporidia carriage among immunocompromised patients hospitalized at a major hospital complex in the Tunis capital area, Tunisia (North Africa), and perform molecular epidemiology and population structure analyses of *Enterocytozoon bieneusi*, which is an emerging fungal pathogen. We screened 250 stool samples for the presence of intestinal microsporidia from 171 patients, including 81 organ transplant recipients, 73 Human Immunodeficiency Virus (HIV)-positive patients, and 17 patients with unspecified immunodeficiency. Using a nested PCR-based diagnostic approach for the detection of *E. bieneusi and Encephalitozoon* spp., we identified 18 microsporidia-positive patients out of 171 (10.5%), among which 17 were infected with *E. bieneusi*. Microsporidia-positive cases displayed chronic diarrhea (17 out of 18), which was associated more with HIV rather than with immunosuppression other than HIV (12 out of 73 versus 6 out of 98, respectively, *p* = 0.02) and correlated with extended hospital stays compared to microsporidia-negative cases (60 versus 19 days on average, respectively; *p* = 0.001). Strikingly, internal transcribed spacer (ITS)-based genotyping of *E. bieneusi* strains revealed high-frequency occurrence of ITS sequences that were identical (*n* = 10) or similar (with one single polymorphic site, *n* = 3) to rare genotype WL12. Minimum-spanning tree analyses segregated the 17 *E. bieneusi* infection cases into four distinct genotypic clusters and confirmed the high prevalence of genotype WL12 in our patient population. Phylogenetic analyses allowed the mapping of all 17 *E. bieneusi* strains to zoonotic group 1 (subgroups 1a and 1b/1c), indicating loose host specificity and raising public health concern. Our study suggests a probable common source of *E. bieneusi* genotype WL12 transmission and prompts the implementation of a wider epidemiological investigation.

## 1. Introduction

Microsporidia are unicellular spore-forming protozoan-like fungi. They are obligate intracellular pathogens colonizing an extremely wide range of other eukaryotes, including both vertebrate and invertebrate hosts [1]. Spores from microsporidia are found in drinking water, wastewater, and recreational waters [2,3,4,5,6], which is in line with the waterborne route as the main transmission route of microsporidia. Consequently, microsporidia are considered as priority pathogens in the emerging infectious diseases/pathogens category B of the National Institute for Allergy and Infectious Diseases classification. The phylum Microsporidia contains more than 200 genera and 1500 species. Of these, 17 are considered pathogenic to humans, particularly *Enterocytozoon bieneusi* (*E*. *bieneusi*) and the three *Encephalitozoon* species *Enc*. *intestinalis*, *Enc*. *hellem*, and *Enc*. *cuniculi* [7].

*E*. *bieneusi* is the most commonly identified agent of chronic diarrhea in immunocompromised individuals, particularly in acquired immunodeficiency syndrome (AIDS) patients, organ transplant recipients, cancer patients undergoing immunosuppressive treatment, and/or patients with acquired or congenital immune system failure [8]. Importantly, Tunisia (population ≈ 11.8 M) is among countries of Middle East and North Africa with the highest incidence rates of HIV/AIDS [9], totaling 1303 diagnosed cases as of December 2020. In 2019, ≈50 patients received bone marrow transplants, while an additional 50 individuals underwent kidney (31 patients, 1492 patients from 1986 to 2015 [10]), heart (7 patients), and liver (10 patients) transplantations in the country. The diagnosis of intestinal microsporidiosis has traditionally depended on the direct visualization of microsporidia spores in infected material—commonly stool specimens—by light microscopy using Weber’s modified trichrome (MT) staining [11]. This common method can be difficult to manage and is not sensitive due to the small size of the spores (1–3 µm) and the variability in the quality of the staining [11]. In addition, this technique does not allow identification to the species level. Moreover, the non-cultivable nature of microsporidia and the nonexistence of an effective culture system made the molecular diagnosis a very reliable and fast tool for their detection. Investigations can be carried out directly from infected biological samples, which usually use DNA from fecal specimens [12]. The development of next-generation sequencing and metagenomics will probably facilitate both the diagnosis of intestinal microsporidiosis in the future [13,14] and the implemention of novel therapeutic strategies based on manipulation of the gut microbiota [15].

Current treatment of microsporidiosis relies on the administration of albendazole, which inhibits microtubule polymerization by binding to tubulin, and fumagillin, which inhibits methionine aminopeptidase type 2; however, albendazole has no demonstrated efficacy against *E*. *bieneusi* [16,17,18]. Consequently, the identification of microsporidia at the species level is essential for an effective drug prescription. Since the 1990s, the development of polymerase chain reaction (PCR)-based methods allowed the rapid detection and species identification of microsporidium germs in patients at risk [12]. This method requires the use of PCR primers targeting the small subunit ribosomal RNA (SSU rRNA) gene and/or the internal transcribed spacer (ITS) region of the ribosomal RNA gene [19].

Analyses of the polymorphism of the ITS nucleotide sequences are also used for the molecular epidemiology analyses of *E*. *bieneusi* infections and for genotyping *E*. *bieneusi* [19,20]. A total of 474 distinctive genotypes have been identified and phylogenetically mapped to 11 major genetic groups, named Group 1 to Group 11 [20]. Group 1 is the largest group, which is subdivided into nine subgroups designated 1a to 1i, and carrying 314 genotypes [20]. Within this group, genotypes found in both humans and wild/domestic animals are frequent (e.g., D, EbpC, and Type IV), pointing to a low level of host specificity and the potential for zoonotic or cross-species transmission. Therefore, such genotypes should be considered of public health importance. Similarly, Group 2 genotypes (subdivided into 2a, 2b, and 2c) can also affect a diverse range of hosts, including humans; however, Groups 3–11 are enriched with genotypes that appear to be host-specific [20]. Although one could provide strong argument in favor of zoonotic or interspecies transmission in case of the co-occurrence of identical *E*. *bieneusi* genotypes among different host species, it is still difficult to ascertain the transmission route and assess the clinical and epidemiological risk factors of *E*. *bieneusi* infections [21]. As stated above, one could reasonably speculate that the transmission route may occur from person to person (anthroponotic transmission), particularly when the genotypes are host specific. On the other hand, animal-to-human transmission (zoonotic transmission) implies other genotypes that infect both humans and animals [21]. Some rare genotypes were classified among those associated with “miscellaneous hosts”, such as genotype WL12 [19], which was initially found in beaver and otter from eastern Maryland in the United States [22]. Genotype WL12 was later on reported in one HIV-infected patient from Brazil [23]; then, it was detected in wastewater samples from China [24] and Tunisia [25], suggesting the potential for zoonotic/waterborne transmission. In this study, we report the high prevalence of *E*. *bieneusi* genotype WL12 among HIV-infected and organ transplant recipient patients from a major hospital complex in Tunis, Tunisia (North Africa).

## 2. Materials and Methods

### 2.1. Study Population and Collection of Fecal Specimens

All procedures complied with ethical standards for human investigations and the principles of the Declaration of Helsinki. The present study protocol was reviewed and approved by the Ethics Committee at La Rabta University Hospital, the Tunisian Ministry of Health, Tunisia (Application file # CEBM.EPS.HR/02/2020). Two hundred and fifty stool samples were collected over a 3-year period (January 2014 to December 2016) from 171 immunocompromised patients at the Bab Saadoun Hospital Complex in Tunis, Tunisia (Table S1). Ninety patients have stayed 21 days on average at the La Rabta Hospital (Division of Infectious Diseases and Kidney Transplantation Unit at the Department of Nephrology, Table S1), 64 patients were hospitalized from 3 to 192 days at the Bone Marrow Transplant Center, and 17 patients with unspecified immunodeficiency were hospitalized from 3 to 34 days at the Salah Azaiez Cancer Center (7 patients) and at the Béchir Hamza Children’s Hospital (10 patients) (Table S1). All patients were screened for microsporidia using parasitological methods. Clinical parameter data were collected from at least 157 patients. All stool samples were analyzed for the detection of microsporidia spores using light microscopy after modified Weber’s trichrome staining [26]. Each slide was examined by two different examiners.

### 2.2. DNA Extraction and PCR Amplification

Stool specimens were stored at −20°C until DNA extraction. Total genomic DNA was extracted using a QIAamp DNA stool mini-kit (QIAGEN, Hilden, Germany) in accordance with the manufacturer’s instructions. The extracted fecal DNA was subjected to amplification of the SSU rRNA conserved region from microsporidia using a two-step nested PCR [27]. For the primary PCR, a PCR product of 1200 bp was amplified using the pan-microsporidian primers C1 and C2 [28] (Table S2). For the secondary PCR, species-specific primers EBIEF1/EBIER were used to amplify a 607-bp fragment from *E*. *bieneusi*; V1/SI500 were used to amplify a 370-bp fragment from *Enc*. *intestinalis*; EHELF/EHELR were used to amplify a 547-bp fragment from *Enc*. *Hellem*; and ECUNF/ECUNR were used to amplify a 549-bp DNA fragment from *Enc*. *cuniculi* (Table S2). All PCR reactions were performed individually for each of the 4 species. In addition, more than one stool sample was tested by nested PCR from 13 out of 18 microsporidia-positive patients (2 to 9 samples per patient, Table S1). The DNA was amplified in a 50-μL reaction mixture containing 12.5 pmol of each primer, 200 mM each of deoxynucleosidetriphosphate, 2 mM MgCl2 and 1U of Taq DNA polymerase (Invitrogen, Waltham, MA, USA). After initial denaturation of the DNA at 94 °C for 15 min, 30 cycles were run as follows: denaturation step at 94 °C for 1 min, annealing step at 56 °C for 1 min, and elongation step at 72 °C for 1 min, with 5 min of final extension at 72 °C after 30 cycles.

### 2.3. Genotyping of E. bieneusi Isolates by ITS Sequencing

Genotyping of *E. bieneusi* isolates was performed by nested-PCR amplification of the entire ITS region (243 bp) [20] using the outer primers EBITS3 and EBITS4 (Table S2) and the inner primers EBITS1 and EBITS2.4 (Table S2). These reactions produced fragments of 435 and 390 bp, respectively. The ITS genotypes of *E. bieneusi* were named according to the established nomenclature [19]. All PCR products were purified and sequenced in both directions according to the Sanger method using an ABI PRISM 377 DNA Sequencer (Applied Biosystems, Foster City, CA, USA). The resulting sequences were analyzed using BioEdit Sequence Alignment Editor Version 7.0.5.3 and compared with equivalent sequences available at the National Center for Biotechnology Information (NCBI) database using the Blastn suite of algorithms (https://blast.ncbi.nlm.nih.gov/Blast.cgi (accessed on 24 February 2021)). To correct for wrongly assigned *E. bieneusi* genotypes on NCBI, we additionally relied on comparison with sequences provided by Li *et al*. (2019, Tables S2–S12) [13], where authors updated the associations between *E. bieneusi* ITS genotypes and NCBI accession numbers/entries available to date. Nucleotide sequences obtained in this study were deposited at the GenBank database under accession numbers MN596813–MN596826 and MW272504-MW272506.

### 2.4. Phylogenetic Analyses

The ITS sequences of the present study were compared to an equivalent set of sequences from previously characterized *E. bieneusi* genotypes identified in humans, animals, and the environment. A phylogenetic analysis was performed through the construction of a neighbor-joining tree using Mega X program version 10.0.5 (http://www.megasoftware.net/ (accessed on 24 February 2021)), Institute for Genomics and Evolutionary Medicine, Temple University, Philadelphia, PA, USA) based on the evolutionary distances calculated by the Kimura-2-parameter model. A bootstrap analysis was used to assess the robustness of the resulting clusters using 1000 replicates. Population structure analyses (i.e., minimum-spanning tree) were performed using Bionumerics version 7.6 (Applied Maths, Sint-Martens-Latem, Belgium).

### 2.5. Statistical Analyses

Chi square (χ2) statistical tests were used for testing associations between two or more categorical variables. For comparing means, we used a Welch’s *t*-test (unequal variances t-test). *p*-values less than 0.05 were considered significant.

## 3. Results

### 3.1. E. bieneusi is the Major Species Infecting Intestinal Microsporidia-Positive Immunocompromised Patients

**Figure 1:**
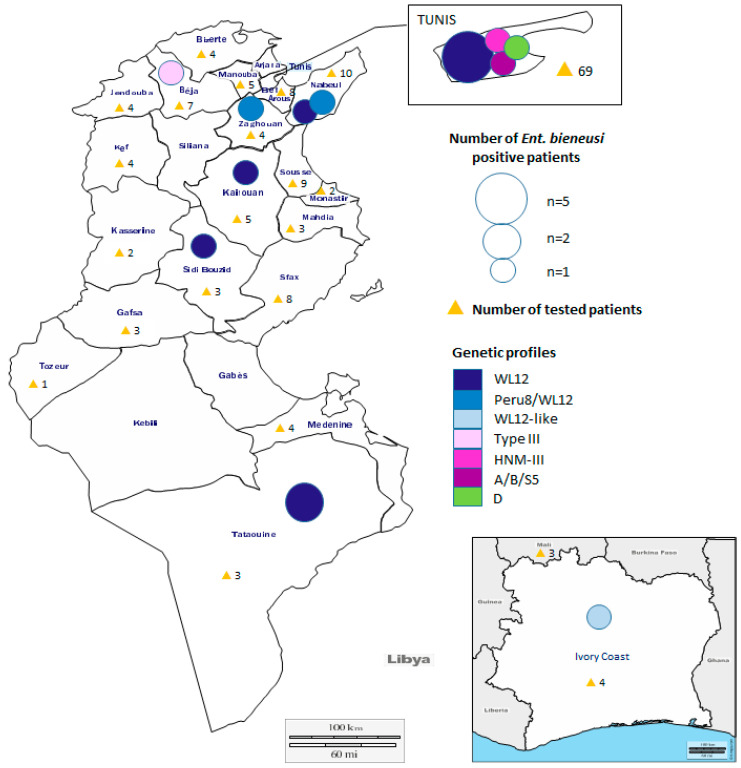
Geographical distribution of the genetic profiles of *E*. *bieneusi* infecting intestinal microsporidia-positive patients. The main map shows the different Tunisian districts/cities where *E*. *bieneusi*-positive patients (colored spheres) reside. The number of recruited patients originating from each district/city is indicated next to yellow triangles. Sphere sizes are proportional to the number of patients infected with *E*. *bieneusi* (*n* = 1 to 5). The color of the spheres was assigned according to the identified genotypes shown on the legend with a color code, entitled “Genetic profiles”. One patient originating from Ivory Coast (bottom left map) carries *E*. *bieneusi* WL12-like genotype.

We recruited 171 immunocompromised (81 organ transplant recipient, 73 HIV-positive, and 17 with unspecified immunodeficiency) patients who have been hospitalized for 23 days on average (3 to 192 days) at the Bab Saadoun Hospital Complex in Tunis capital (Tunisia, North Africa) from January 2014 to December 2016. One hundred and fifty-eight patients originated from various Tunisian districts/cities (Figure 1), whereas 13 others were immigrants from the Ivory Coast (4 patients), Mali (3 patients), Cameroon (3 patients), Congo (2 patients), and Mauritania (1 patient) (Figure 1, Table S1). Ninety patients have stayed from 5 to 101 days at the La Rabta Hospital, 64 patients were hospitalized from 3 to 192 days at the Bone Marrow Transplant Center, and 17 patients with unspecified immunodeficiency were hospitalized from 3 to 34 days at the Salah Azaiez Cancer Center (7 patients) as well as at the Béchir Hamza Children’s Hospital (10 patients).

Using nested PCR reactions from 250 fecal DNA extracts (see Materials and Methods, Table S2), we identified 18 microsporidia-positive cases out of all 171 patients (10.5%). Of these, 17 suffered from chronic diarrhea, which significantly correlated with the presence of intestinal microsporidia (Table 1). 

We found that microsporidia-positive cases associated more with HIV infection (*n* = 12 out of 73, 16.4%) rather than with immunodeficiency other than HIV (*n* = 6 out of 98, 6.1%; χ2 = 4.72, *p* = 0.03) (Table 1); and they were more often observed at the La Rabta Hospital (14 cases out of 90; χ2 = 5.10, *p* = 0.02) rather than at the Bone Marrow Transplant Center, the Salah Azaiez Cancer Center or Children’s Hospital (4 cases out of 81) (Table S1). In addition, microsporidia-positive patients had significantly longer hospital stays (60 days on average, *n* = 18) than microsporidia-negative patients (19 days on average, *n* = 153, *p* = 0.001 using a Welch’s *t*-test).

Importantly, 17 out of the 18 microsporidia-positive cases were infected with *E. bieneusi*, including one patient co-infected with *Enc. intestinalis* and *Enc. cuniculi* (Table S1). Microsporidia spores were not regularly detected after modified Weber’s trichrome staining, reflecting the advantage of the molecular diagnosis approach.

### 3.2. ITS Sequencing Reveals High-Frequency Occurrence of E. Bieneusi Genotype WL12

To better understand the molecular epidemiology of *E. bieneusi* infection/transmission in our patient population, *E. bieneusi*-positive stool DNA samples were used to perform nested PCR reactions and amplify the full ITS region using the outer primer pair EBITS3 and EBITS4 and the inner primer pair EBITS1 and EBITS2.4 (Table S2, [20]). We bidirectionally sequenced all 17 amplified ITS fragments and performed a Basic Local Alignment Search Tool (BLAST) analysis at the NCBI gene bank to assign genotypes to each of the 17 *E. bieneusi*-positive samples (see Materials and Methods). Strikingly, 10 ITS sequences out of 17 were identical to equivalent sequences from genotype WL12 (Table 2, Figure 1), while two additional sequences differed from the WL12 or the Peru8 genotypes only by one pyrimidine-to-pyrimidine polymorphism (Table 2). The remaining sequences were assigned genotypes D (patient 94), HNM-III (patient 34, identical to HNM-III except C137→T polymorphism), Type III (patient 37, identical to Type III except C97→T polymorphism), A/B/S5 (identical to A, B and S5 except G31→A, A77→G and C137→T polymorphisms, respectively) from patient 9 and a genotype from Ivorian patient 152 that differs from WL12 in a pyrimidine-to-purine substitution at position 137 (WL12-like, C137→G) (Table 2).

The identified genotypes differ in the seven polymorphic positions 31, 93, 97, 117, 134, 137, and 178 with respect to the first nucleotide of the full 243-bp ITS region (Table 3).

**Figure 2:**
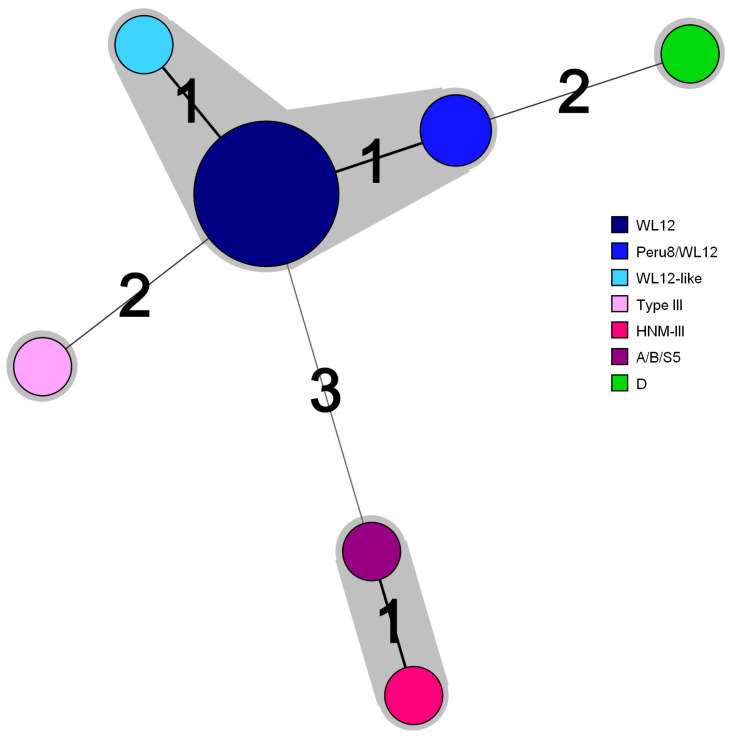
Minimum-spanning tree profile of ITS sequences from *E. bieneusi* strains infecting intestinal microsporidia-positive patients. Each node corresponds to a distinct genotype, indicated on the legend with a color code (right side). The size of the spheres is proportional to the number of strains sharing the same genotype. The shaded zones (light gray) indicate clusters of similar ITS genotypes (i.e., ITS sequences with no more than one nucleotide difference). Numerals connecting the circles indicate the number of single nucleotide polymorphisms that differentiate a given genotype from another.

To better visualize the extent of similarity/divergence between the identified genotypes, we constructed a minimum-spanning tree (see Materials and Methods, Figure 2). As expected, genotypes WL12 (*n* = 10), Peru8/WL12 (*n* = 2), and WL12-like (*n* = 1) clustered together, infecting in total 13 patients out of 17 (blue spheres, Figure 2). Genotypes D and Type III formed singletons (pink and green spheres, Figure 2), whereas genotypes HNM-III and A/B/S5 were more distantly related to WL12, forming a cluster that differs by at least three substitutions (brown and purple spheres, Figure 2). Our minimum-spanning tree analysis revealed that the 17 *E. bieneusi* infection cases were associated with four distinct genotypic clusters and confirms the high prevalence of genotype WL12 in our patient population.

### 3.3. Phylogenetic Analyses Assign all *E. bieneusi* Strains to Zoonotic Group 1

**Figure 3:**
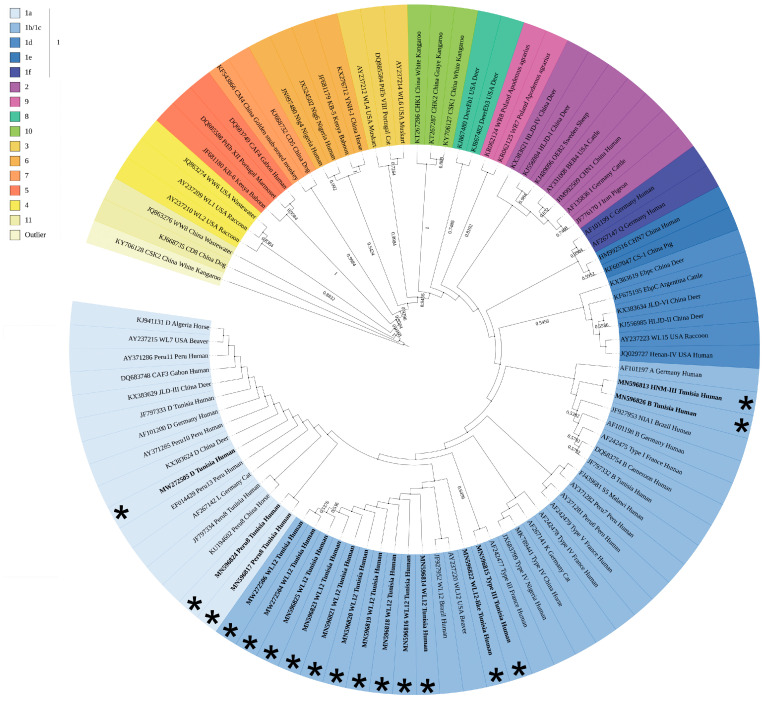
Phylogenetic relationship of the 17 *E. bieneusi* strains infecting our patient population with a selection of previously published *E. bieneusi* ITS genotypes representative of each genetic group. The 11 genetic groups and the outlier are indicated with a color code shown on the legend (top left). Bootstrap values are shown on the edges of the tree. The 17 *E. bieneusi* strains from our patient population are shown in bold and indicated with asterisks. The tree was constructed using a neighbor-joining analysis of the ITS sequences based on the evolutionary distances calculated by the Kimura two-parameter model.

To further resolve the genetic relatedness of *E. bieneusi* strains infecting all 17 patients and map them to phylogenetic groups, we constructed a neighbor-joining tree of the ITS sequences identified in this study and included equivalent sequences from representative genotypes from each of the 11 genetic groups reported so far [20] (Figure 3). The highly divergent ITS sequence from *E. bieneusi* genotype CSK2 was used as an outgroup [20]. All genotypes fell into zoonotic group 1 (Figure 3, clusters in shades of blue). The Peru8/WL12-related strains from patients 11 (MN596824) and 56 (MN596817) and genotype D from patient 94 (MW272505) clustered among genotypes from subgroup 1a (Figure 3, pale blue), whereas the remaining genotypes (WL12, HNM-III, Type III and WL12-like) clustered among those from subgroups 1b/1c (Figure 3, darker blue). Our phylogenetic analysis suggests that *E. bieneusi*-infected patients carry strains with the potential for zoonotic or cross-species transmission and emphasizes the public health importance of the diagnosed cases.

## 4. Discussion

The growing interest in research on microsporidia began since the 1980s, with the emergence of the HIV/AIDS epidemic, as microsporidia is responsible for severe chronic diarrhea among HIV-infected patients [29,30,31]. There has been a growing number of microsporidia infection cases among patients undergoing organ transplantation as well, most of them displaying chronic diarrhea [32,33,34]. Here, we found that diarrhea strongly correlated with intestinal microsporidia carriage (Table 1), but we could not determine whether it is a direct consequence of microsporidiosis per se (i.e., symptomatic infection), which is one limitation of our study. Additionally, as all of the patients were immunocompromised and they are a risk group of different opportunistic infections, diarrhea could be due to the presence of co-infection with pathogens other than microsporidia or to graft-versus-host disease (GvHD) in the case of transplant recipients. Following parasitological examination of the feces, we found three cases of *Dientamoeba fragilis* carriage in patients #80, #94, and #107. We also performed a modified Ziehl–Neelsen staining for detecting *Cryptosporidium* spp, and two HIV patients were positive for *Cryptosporidium* oocysts (patients #38 and 152). Among these five patients, only patients 94 and 152 are positive for microsporidia. Unfortunately, methods other than microscopy (e.g., molecular methods) were not used to further screen for co-infection cases. Since no information on follow up with regard to microsporidia-specific treatment—if any—could be obtained, it is currently unknown whether patients with diarrhea became symptom-free after treatment, which could have pointed to a direct microsporidia-diarrhea cause–effect relationship. Likewise, attempts for information on GvHD cases were unsuccessful.

In Tunisia and other countries of the region, microsporidia were, and still are, often overlooked and misdiagnosed because they are not specifically identified in most medical diagnostic laboratories. The recourse to staining techniques, such as the Modified Weber Trichrome staining, usually results in poor sensitivity that varied from 25% to 79% [35,36]. The low sensitivity of the mentioned method can explain their irregular detection in our hands. Thereby, the diagnosis was confirmed on the positivity of the PCR reaction and declared positive in 18 patients out of 171, pointing to a prevalence of 10.5%. Depending on many factors, including the immunological status of the population under study, geographical origin, and diagnosis methods, the prevalence of microsporidia infections can reach up to 50% [37,38,39]. Wang et al. estimated that microsporidia prevalence varied largely between countries/continents. In HIV-infected patients, it was predicted around 15.4% in sub-Saharan Africa, 14.4% in western and central Europe and North America, 11.7% in Asia and the Pacific, 5.6% in Latin America and the Caribbean, 2.2% in the Middle East and North Africa, and 13.0% in eastern Europe and central Asia [39]. The prevalence of microsporidia among HIV-infected patients appears to be increasing in Tunisia, as we report here a higher prevalence (16.4%) compared to those reported previously in the country (11.7% in 2009 [40], 15.7% in 2017 [41]).

The most significant manifestation of microsporidiosis, from the perspective of public health, is gastrointestinal tract infection in patients with HIV/AIDS. Our study is one of the few studies that investigated microsporidia carriage among patients with immunodeficiency other than HIV/AIDS, i.e., bone marrow transplant, kidney transplant recipients, or those with acquired or congenital immune system failure, reporting six cases out of 98 patients (6.12%, Table S1). Most of the microsporidia cases described so far from renal, liver, and hematopoietic transplant recipients were of digestive origin [42,43,44,45,46,47,48], with a prevalence ranging from 1% to 25%, while a few cases reported urinary [49] as well as respiratory [50,51] infections. Although the proportion of immunocompromised patients other than those infected with HIV was higher in our population—i.e., *n* = 98 (57.3%), versus *n* = 73 patients with HIV (42.7%)—we observed that microsporidia infections associated more with HIV rather than with immunodeficiency other than HIV (*p* = 0.03, Table 1). The observation that most of the microsporidia cases were often diagnosed at the La Rabta Hospital (*p* = 0.02) is explained by the fact that it hosted all HIV-infected patients (Table S1). A study conducted in Portugal between 1999 and 2009, involving 856 patients, showed that *E. bieneusi* was more common in the HIV-seropositive group of patients than in the HIV-seronegative group, the latter including nine patients with other causes of immunosuppression [52]. It is unclear as to whether the type of immunodeficiency disorder influences the susceptibility to microsporidiosis and/or infection with microsporidia. Infections are usually accompanied by chronic carriage of the pathogen [53], implying the occurrence of dynamic microsporidia–host interactions whereby both innate and adaptive immune responses operate. Studies using *Encephalitozoon* spp. infection models in mice suggest that both CD4^+^ and CD8^+^ cells play a protective role, CD4^+^ cells being involved in response to natural route of infection (i.e., oral infection model) [54,55]; while CD8^+^ are required for protecting mice against intraperitoneal infection [56]. In fact, more studies pointed to a complex interplay between CD4^+^ and CD8^+^ cells, which also implicates mediators of immune functions, such as IFN-γ and IL-2, in addition to specific immune checkpoint receptors (e.g., killer-cell lectin like receptor G1) and other immune cell types, including dendritic cells, γδ T cells, and B-1 cells [57]. Collectively, more mechanistic investigations are needed to further understand the pathophysiological/immunological aspects pertaining to the pathogenesis of microsporidiosis in patients at risk and whether these aspects could explain the variability in terms of microsporidiosis-susceptibility patterns depending on the clinical context.

It is striking that WL12, a rarely observed genotype, occurs so frequently among *E. bieneusi* strains infecting our patient population (Figure 1). The fact that genotype WL12 has been previously reported among wild animals [22], in a patient with HIV [23], and from wastewater samples [24,25] suggests a loose host specificity and increases its importance for public health. The *E. bieneusi* genotype WL12-positive patients reside in various Tunisian districts/cities and are not related to each other (Figure 1, Table S1). A deeper search of the literature allowed us to notice the occurrence of genotype WL12 in wastewater samples from Tunisia [25]. Consequently, we performed an extensive analysis of the Ben Ayed et al. data, where the authors detected the WL12 genotype in raw/treated wastewater from seven water treatment plants out of 18 in different areas of the country, including the Tunis capital area [25]. Since Tunisia is experiencing a growing shortage of water (due to irregular or decreased rainfall), the reuse of treated wastewater has been extensively implemented in agriculture, which may facilitate the dissemination of parasites, including *E. bieneusi*. This indicates that *E. bieneusi* from reused treated wastewater could be a potential source of transmission in Tunisia, and it may explain the high occurrence of genotype WL12 in our patient samples. We further examined the available literature from Tunisia to search for previously reported cases of *E. bieneusi* among Tunisian patients. We found another study dating back to 2012, reporting the detection of *E. bieneusi* in HIV-infected patients [35]. We extracted all 23 *E. bieneusi* ITS sequences available from these published studies at NCBI and generated a phylogenetic tree (Figure S1). We found that WL12 clustered with genotypes Peru8, B, and D identified by Chabchoub et al. from HIV patients as well as with genotypes Type IV and CHN15 identified by Ben Ayed et al. from water treatment facilities (Figure S1). This indicates that a subset of *E. bieneusi* strains isolated from Tunisian patients and from reused treated water in Tunisia belongs to a genetic group with the potential for zoonotic/waterborne transmission. Indeed, in addition to WL12, Ben Ayed et al. also detected genotypes Type IV, Peru8, and D from raw/treated wastewater [25]; all of them were reported in the study by Chabchoub et al. [35].

One could also speculate that transmission might have occurred during the significantly longer hospitalization periods of the microsporidia-infected patients (60 days on average for microsporidia-positive patients versus 19 days on average for non-infected patients, *p* = 0.001, Table S1). This hypothesis is attractive even though longer stays might be linked to other underlying health condition(s). Patient-to-patient transmission in the hospital is unlikely to happen, since there was no significant overlap of hospitalization periods (Table S1). It is tempting to speculate that a common source of contamination at the hospital complex area might be responsible for propagating the WL12 genotype, such as food, beverage, and/or use of contaminated utility areas (e.g., restrooms). Since our study is a retrospective investigation, it is difficult to verify whether food/beverage and common utility areas at the hospital were contaminated prior to patient stool sampling. Nosocomial *E. bieneusi* infections are likely to occur, as suggested in a recent study from a hematology unit of a hospital in France [58]. The authors suggested that a common source of infection at the hospital might be responsible for the propagation of an *E. bieneusi* strain with a novel genotype among three patients with hematological malignancies [58].

We show that all *E*. *bieneusi* genotypes mapped to zoonotic group 1 (Figure 3), indicating the likelihood for zoonotic or waterborne transmission. There is still no direct evidence linking human infections to *E*. *bieneusi* of animal origin; however, frequent contact of humans with household/domestic animals has been considered a significant risk factor for zoonotic transmission [59,60]. Since genotype WL12 was detected in *E*. *bieneusi*-positive wastewater/treated-water samples from Tunisia [25], one could not rule out the occurrence of waterborne transmission through the ingestion of spores from animal origin that contaminate water sources/surfaces. *E*. *bieneusi* transmission can also occur through the anthroponotic route, as described previously [61,62]. For example, both divergent genotypes A/B/S5 and HNM-III (Figure 2), which actually differ from each other only by one pyrimidine-to-pyrimidine polymorphism at position 93 (Table 3), clustered among anthroponotic genotypes A and B (Figure 3). This suggests that patients #9 and #34 carry strains with the potential for person-to-person transmission, although genotype HNM-III has only recently been identified among captive macaques (*Macaca fascicularis*) [63]. Collectively, our study highlights the need for conducting a wider epidemiological investigation and prompts the implementation of typing approaches with increased discriminatory power (e.g., microsatellite analysis). Given the importance of this emerging infectious disease, the relevance of our investigation to public health, and the novelty of our findings with regard to the molecular epidemiology and population structure analyses of *E. bieneusi*, future studies involving a larger number of isolates from animal, environmental, and human origins will allow better resolving the differences between *E*. *bieneusi* strains and more precisely determine the possible sources of contamination.

## Figures and Tables

**Table 1:** Distribution of patients tested for intestinal microsporidia carriage according to clinical parameters (age, gender, underlying immunodeficiency, and presence/absence of diarrhea).

Clinical Parameters		Patients with Microsporidia (*n* = 18)	Patients without Microsporidia (*n* = 153)	*p*-Value *^a^*
**Age *^b^***	1–15 years	3	43	>0.05
	16–35 years	8	37	
	>35 years	6	61	
Gender	Male	7	92	>0.05
	Female	11	61	
Underlying immunodeficiency	HIV+	12	61	<0.05
	Other than HIV (Bone marrow/kidney transplant, unspecified)	6	92	
Diarrhea		17	43	<0.05

***^a^****p*-value was calculated using a χ^2^ test. ***^b^*** Data on age available in 157 patients only.

**Table 2:** Genotypic portrait of *E. bieneusi* infecting 14 microsporidia-positive immunocompromised patients.

Patient# *^a^*	Accession Number *^b^*	Underlying Disease *^c^*	ITS Genotype *^d^*	Identical or Similar Genotypes *^e^*
Patient 34	MN596813	HIV infection	HNM-III	**HNM-III(C137/T):** MK139696
Patient 23	MN596814	Bone marrow transplant	WL12	**WL12:** JF927952, AY237220
Patient 37	MN596815	Kidney transplant	Type III	**Type III(C97/T):** AF242477
Patient 55	MN596816	Bone marrow transplant	WL12	**WL12:** JF927952, AY237220
Patient 56	MN596817	Kidney transplant	Peru8/WL12	**Peru8(C97/T):** MN747470, MH714713, MF476880, KU194602, KT267285, KJ668721, KF305584, KF261771, JX683807, JQ029733, JF927959, JF797334, AY371283, JF909995; **WL12(T93/C):** JF927952, AY237220
Patient 74	MN596818	HIV infection	WL12	**WL12:** JF927952, AY237220
Patient 85	MN596819	Bone marrow transplant	WL12	**WL12:** JF927952, AY237220
Patient 87	MN596820	HIV infection	WL12	**WL12:** JF927952, AY237220
Patient 139	MN596821	HIV infection	WL12	**WL12:** JF927952, AY237220
Patient 152	MN596822	HIV infection	WL12-like	**WL12(C137/G):** JF927952, AY237220
Patient 138	MN596823	HIV infection	WL12	**WL12:** JF927952, AY237220
Patient 11	MN596824	HIV infection	Peru8/WL12	**Peru8(C97/T):** MN747470, MH714713, MF476880, KU194602, KT267285, KJ668721, KF305584, KF261771, JX683807, JQ029733, JF927959, JF797334, AY371283, JF909995; **WL12(T93/C):** JF927952, AY237220
Patient 49	MN596825	HIV infection	WL12	**WL12:** JF927952, AY237220
Patient 9	MN596826	HIV infection	A/B/S5	**A(G31/A):** MK982500, MN136776, MH500238, KU886089, KP325475, KP325476, JQ437574, AY371276, AY357185, AY168419, AF101197; **B(A77/G):** AF242475, AF101198, JF797332; **S5(C137/T):** MG458710, FJ439681, DQ885581
Patient 15	MW272504	HIV infection	WL12	**WL12:** JF927952, AY237220
Patient 94	MW272505	HIV infection	D	**D:** MN704918, MN747468, MK982516, MN902234, others
Patient 121	MW272506	HIV infection	WL12	**WL12:** JF927952, AY237220

***^a^*** Each patient was assigned an identification number according to the chronology of sampling (see Table S1 for a complete description); ***^b^*** NCBI accession number for each *E. bieneusi* internal transcribed spacer (ITS) sequence identified in this study. ***^c^*** Underlying disease for each *E. bieneusi*-positive patient according to the information provided in Table S1. ***^d^*** ITS genotype was assigned following a BLAST analysis of each ITS sequence against those available at the NCBI database. ***^e^*** The NCBI accession numbers of identical or similar genotypes (indicated in bold) are provided. For similar genotypes, only one single nucleotide polymorphism (shown between parentheses) differentiates the genotypes identified in this study from the known ones.

**Table 3:** Nucleotide variation in seven polymorphic sites of the *E. bieneusi* ITS region from 17 *E. bieneusi*-positive samples.

Number of Patients	Genotype		Nucleotide Position *^a^*					
		31	93	97	117	134	137	178
10	WL12	G	T	T	G	C	C	G
2	Peru8/WL12		C					
1	WL12-like						G	
1	HNM-III	A	C	C			T	
1	A/B/S5	A		C			T	
1	Type III					T		A
1	D		C	C	T			

***^a^*** Nucleotide position, in bp, relative to the first nucleotide of the entire 243-bp ITS fragment.

## Data Availability

The data presented in this study are openly available in NCBI at https://www.ncbi.nlm.nih.gov/nucleotide/under (accessed on 24 February 2021) accession numbers MN596813–MN596826 and MW272504-MW272506.

## Supplementary Materials

The following are available online at https://www.mdpi.com/2309-608X/7/3/161/s1, Figure S1: Phylogenetic tree of *E*. *bieneusi* strains from Tunisia, Table S1: List of patients recruited in this study, Table S2: Primers used in this study.
