# Supplementary material for: High Frequency of Enterocytozoon bieneusi Genotype WL12 Occurrence among Immunocompromised Patients with Intestinal Microsporidiosis
Source: J Fungi (Basel). 2021 Feb 24;7(3):161. doi: 10.3390/jof7030161 (PMC7996336; doi:10.3390/jof7030161)
Supplement: Supplementary file 1 [file jof-07-00161-s001.zip › Figure S1.pdf]

# High Frequency of *Enterocytozoon bieneusi* Genotype WL12 Occurrence among Immunocompromized Patients with Intestinal Microsporidiosis.

Supplementary Material

Figure S1

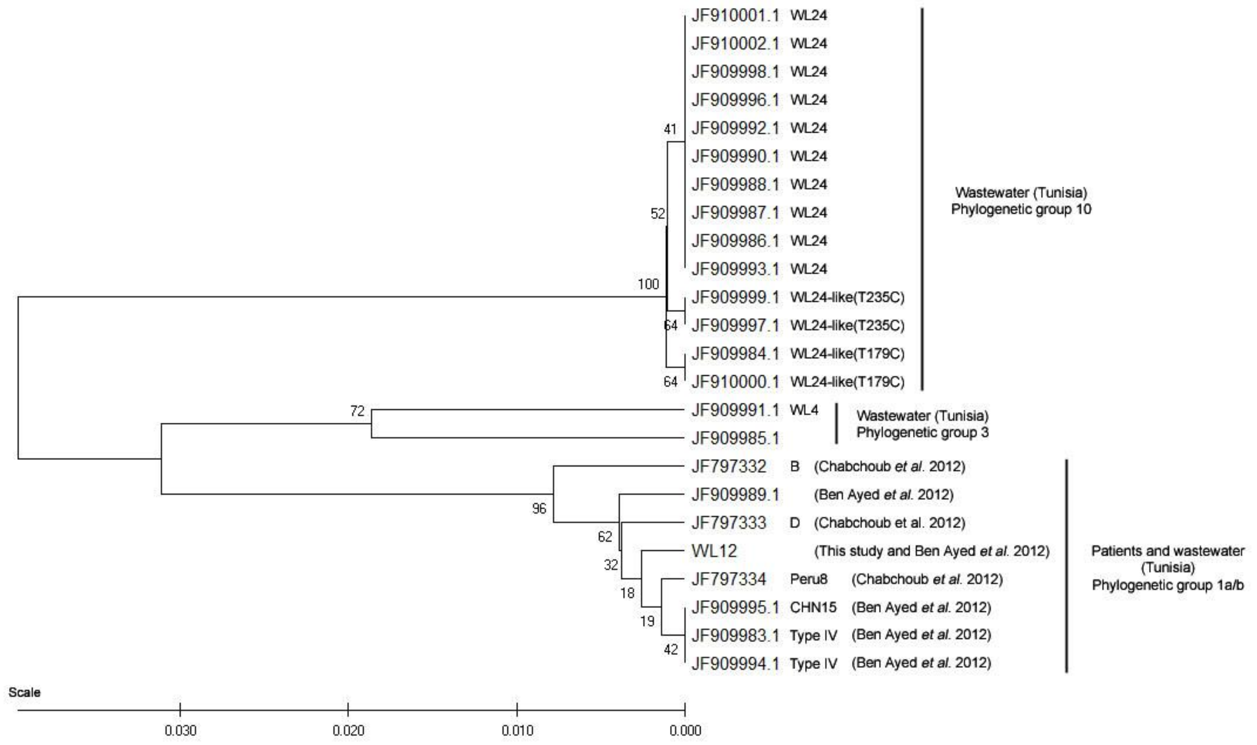


Figure S1. Phylogenetic tree of *E. bieneusi* strains from Tunisia. Tree with ITS sequences (243 bp each) from this study (WL12), the Ben Ayed *et al*. study (20 sequences) and the Chabchoub *et al*. study (3 sequences) was inferred using the UPGMA method. The percentages of replicate trees in which the associated ITS sequences clustered together in the bootstrap test (1000 replicates) are shown next to the branches. The tree is drawn to scale, with branch lengths in the same units as those of the evolutionary distances used to infer the phylogenetic tree. The evolutionary distances were computed using the Kimura 2-parameter method and are in the units of the number of base substitutions per site. Evolutionary analyses were conducted with the Mega X software (See Materials and Methods).
